# Associations between cardiovascular health and low thyroid function among US adults: a population-based study

**DOI:** 10.3389/fendo.2024.1437386

**Published:** 2024-09-27

**Authors:** Xiaoxiao Fang, Rui Hu, Shani Fei, Zhiguo Ding, Jiuli Zhao, Jianwei Shang

**Affiliations:** ^1^ Dongzhimen Hospital, Beijing University of Chinese Medicine, Beijing, China; ^2^ Graduate School of Beijing University of Chinese Medicine, Beijing, China; ^3^ Department of Thyropathy, Sunsimiao Hospital, Beijing University of Chinese Medicine, Tongchuan, Shaanxi, China

**Keywords:** low thyroid function, cardiovascular health, life’s essential 8, NHANES, cross-sectional study

## Abstract

**Background:**

Higher thyroid-stimulating hormone (TSH) amidst normal thyroid hormone (TH) levels may contribute to a negative impact on cardiovascular health (CVH). We sought to probe the associations between Life’s Essential 8 (LE8), a newly revised CVH evaluation, and low thyroid function among US adults.

**Methods:**

The datasets from the 2007-2012 National Health and Nutrition Examination Survey (NHANES) were applied to the study. Low-normal thyroid function and subclinical hypothyroidism (SCH) were both regarded to be low thyroid function. Multivariable logistic regressions were utilized to inquire about the relationship between LE8 and low thyroid function.

**Results:**

Among the 6,315 participants (age ≥20 years), 1,375 (21.77%) were ascertained to be low thyroid function. After adjusting possible confounders, a higher LE8 score was linked to a lower probability of experiencing low thyroid function (Odds ratio [OR] for each 10-point increase: 0.923 [95% CI, 0.884-0.964]). A similar correlation was found between the health factors score and low thyroid function (OR for each 10-point increase: 0.905 [95% CI, 0.876-0.935]). Also, scoring better on physical activity (PA), body mass index (BMI), blood lipid, blood glucose (BG), and blood pressure (BP) may be conducive to reducing the rates of low thyroid function. Furthermore, subgroup and sensitivity analyses indicated that the negative correlations were generally robust.

**Conclusions:**

The LE8 score and health factors score were nonlinearly and negatively related to the prevalence concerning low thyroid function. Promoting the regulation of optimum CVH levels could work on mitigating the load of low thyroid function and cardiovascular diseases (CVDs).

## 1 Introduction

Low thyroid function, a condition of high TSH level in the presence of normal TH level, comprises low-normal thyroid function and SCH (1). TH receptors are situated in myocardial and vascular endothelial organizations, thus slight variations in TH can alter cardiovascular function and affect end-organ regulation (2, 3). It is now commonly recognized that significant hypothyroidism adversely contributes to the morbidity and mortality of CVDs (4). However, a growing body of evidence implies that low thyroid function may also compromise cardiometabolic capacity, dramatically enhancing the danger of hypertension, atherosclerosis, arrhythmias, and other CVDs (5, 6). Meanwhile, it has been anticipated that 19.05 million deaths of CVD worldwide in 2020, which represents an increase of 18.71% from 2010 (7). As a result, proactive screening and monitoring of CVDs in low thyroid function patients may be of assistance in improving CVH.

The American Heart Association (AHA) launched Life’s Simple 7 (LS7) in 2010, which includes 3 health behaviors and 4 health factors to better surveillance of the CVH status of the general population (8). In 2022, in response to the desire to enhance feasibility in practice, the AHA, after more than a decade of accumulated evidence and inspiration, updated the CVH quantitative assessment instrument, known as LE8, to circumvent the limitations of LS7 (9, 10). Compared with the original LS7, LE8 has upgraded the scoring specifications of CVH indicators to refine and continuously track the CVH of individuals (11, 12). Additionally, LE8 further incorporated sleep health to capture the essential role of sleep in human life maintenance and cardiometabolic health (7). Taking into account the close connection between low thyroid function and CVDs, promoting CVH may represent an appropriate means of mitigating thyroid dysfunction and CVD damage. There have been no available studies linking LE8 to low thyroid function. This nationwide representative research evaluated the relationship between the two using NHANES data, aiming to generate novel strategies for the long-term management of low thyroid function and CVDs.

## 2 Methods

### 2.1 Study participants

NHANES is a population-based study employing a stratified, sophisticated, and random sampling scheme to deliver a wealth of details about the condition of general health and nutrition in the US. The NHANES study protocol was authorized and affirmed by the National Center for Health Statistics (NCHS) Research Ethics Review Board, and written informed permissions were provided. Public access to more thorough research methodologies and figures is available at https://www.cdc.gov/nchs/nhanes/.

**Figure 1:**
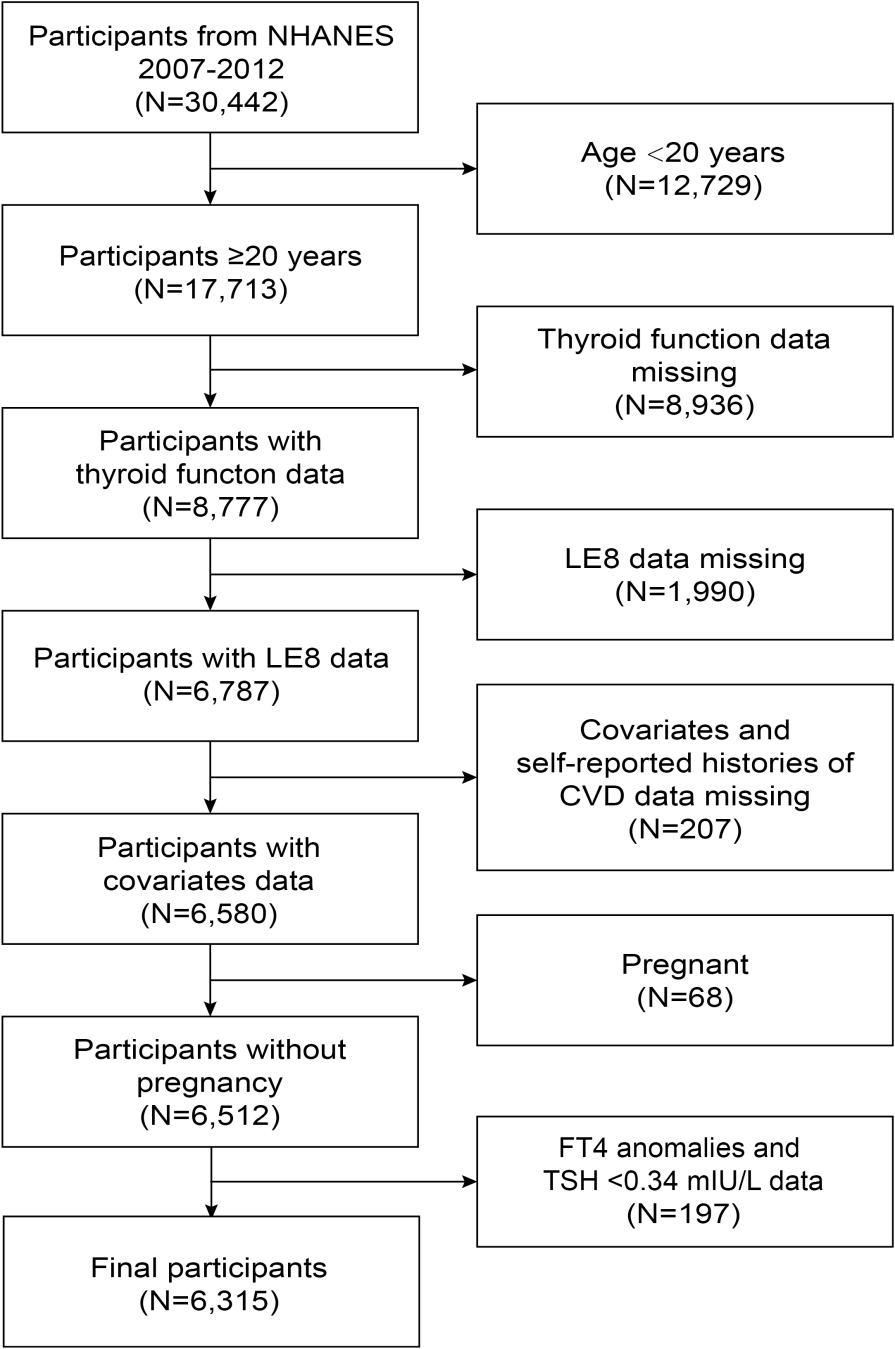
Flowchart of the participant selection from NHANES 2007-2012. NHANES, National Health and Nutrition Examination Survey; FT4, free thyroxine; TSH, thyroid-stimulating hormone; LE8, Life’s Essential 8; CVD, cardiovascular disease.

NHANES data from 2007 to 2012 were used in this study. Among the 30,442 participants, 17,713 were ≥20 years. We eliminated 8,936 participants with missing thyroid function data (TSH, FT4), 1,990 participants with missing LE8 data, 207 participants with missing covariates and self-reported histories of CVD data, and 68 pregnant participants. Additionally, 197 participants with FT4 anomalies and TSH <0.34 mIU/L were also removed. In the end, 6,315 participants were incorporated into the research (**Figure 1**).

### 2.2 Definition and measurement of low thyroid function

The thyroid function parameters investigated in this study contained FT4 and TSH. FT4 was determined by a two-step enzyme immunoassay standardized to a range of 0.6-1.6 ng/dL, while TSH was assayed utilizing a 3rd generation, two-site immunoenzymatic “sandwich” assay standardized to a value of 0.34-5.6 mIU/L. Strict-normal thyroid function was recognized to be a TSH level of 0.34-2.5 mIU/L and a regular FT4 level. Low thyroid function was viewed to be a TSH level over 2.5 mIU/L and a regular FT4 level, including both low-normal thyroid function and SCH (13).

### 2.3 Measurement of LE8

The LE8 score is made up of 4 health behaviors (diet, PA, nicotine exposure, and sleep health) and 4 health factors (BMI, blood lipids, BG, and BP). The elaboration on the computation of the LE8 score utilizing NHANES data was described in **Supplementary Table 1** (9, 14, 15). The Healthy Eating Index-2015 (HEI-2015) was employed for the assessment of dietary metrics, which was computed utilizing data from two 24-hour dietary recall interviews (16). **Supplementary Table 2** outlined the elements and grading criteria for the HEI-2015 (17). Information on the other components of the LE8 came from self-report questionnaires, medical checkups, and gathered blood samples. Each LE8 metric was awarded points from 0 to 100. The overall LE8 score was determined by the unweighted average of the 8 indicators. The AHA proposed quantifying CVH depending on the LE8 score, with 80-100, 50-79, and 0-49 representing high, moderate, and low CVH, respectively (9).

### 2.4 Study covariates

Potential confounders associated with Life’s Essential 8 and low thyroid function were integrated into the final statistical analyses following previous studies (18, 19). The covariates comprised age, sex, race/ethnicity, education level, marital status, and urine iodine concentration (UIC). For these, age was categorized as <65 years and ≥65 years. Race/ethnicity was stratified into non-Hispanic White, non-Hispanic Black, Mexican American, other Hispanic, and Other races. Education level was divided into <high school, high school, and ≥high school. Marital status was separated into three types: married/living with a partner, divorced/separated/widowed, and never married. UIC was dichotomized into <100 ug/L, 100-300 ug/L, and ≥300 ug/L. In addition, this investigation demonstrated the prevalence of diabetes and hypertension among the participants. Diabetes was described as a self-reported diagnosis of diabetes, use of insulin or diabetes medications, glycosylated hemoglobin ≥6.5%, fasting blood glucose ≥126 mg/dL, or two-hour postprandial blood glucose ≥200 mg/dL from an oral glucose tolerance test (20). Also, hypertension was identified as a self-reported diagnosis of hypertension, use of hypertensive medications, systolic blood pressure ≥140 mmHg, or diastolic blood pressure ≥90 mmHg (21).

### 2.5 Statistical analysis

The statistical analyses were undertaken using R (version 4.2.3) and EmpowerStats (version 2.0). Participants were organized into two groups according to their TSH status. We used Student’s t-tests for continuous variables that conformed to a normal distribution, nonparametric tests for continuous variables that were not normally distributed, and chi-square tests to characterize baseline characteristics for categorical variables, which were expressed as mean ± standard deviation (SD) or median (interquartile range) for continuous variables, and frequency (percentage) for categorical variables. Multivariable logistic regressions were used to explore the correlation between LE8 and its components with low thyroid function by aligning latent confounders. To ensure that the model does not suffer from the problem of multicollinearity, we included covariates with variance inflation factor (VIF) < 5 in the models. To conduct additional research on the correlation between LE8 and low thyroid function among various cohorts, stratified analyses were performed. Furthermore, we eliminated those who had self-reported histories of CVDs (comprising congestive heart failure, coronary heart disease, angina, and heart attack, N=508) to evaluate the reliability of the outcomes. Then, smoothed curve fitting and threshold effect analyses were carried out. A P-value <0.05 was determined to be statistically significant.

## 3 Results

### 3.1 Baseline characteristics of participants

There were 6,315 adults recruited for this research. **Table 1** summarizes the baseline characteristics of those individuals categorized as to the thyroid function. The mean ± SD age was 50.23 ± 17.65 years, of which 3,162 (50.07%) participants were females. The mean ± SD LE8 score was 64.80 ± 14.94. The number of participants with low, moderate, and high CVH was 1,031 (16.33%), 4,204 (66.57%), and 1,080 (17.10%), respectively. 1,375 (21.77%) participants were considered to have low thyroid function, much more probably as older, non-Hispanic white, married/living with a partner, with a higher UIC, and in greater prevalence of histories of diabetes, hypertension, and CVDs. Compared to those with low thyroid function, participants with strict-normal thyroid function scored higher on LE8, PA, BMI, blood lipids, BG, and BP.

**Table 1:** Baseline characteristics of the NHANES 2007–2012 study participants (n=6,315) based on TSH status.

Characteristics	Overall (n = 6,315)	Strict-normal thyroid function (n=4,940)	Low thyroid function (n=1,375)	*P*-value
**Age, years**	50.23 ± 17.65	48.87 ± 17.37	55.14 ± 17.79	<0.001
**Age strata, n (%)**				<0.001
<65 years	4,753 (75.27%)	3,862 (78.18%)	891 (64.80%)	
≥65 years	1,562 (24.73%)	1,078 (21.82%)	484 (35.20%)	
**Sex, n (%)**				0.093
Male	3,153 (49.93%)	2,494 (50.49%)	659 (47.93%)	
Female	3,162 (50.07%)	2,446 (49.51%)	716 (52.07%)	
**Race/Ethnicity, n (%)**				<0.001
Non-Hispanic White	3,050 (48.30%)	2,236 (45.26%)	814 (59.20%)	
Non-Hispanic Black	1,231 (19.49%)	1,074 (21.74%)	157 (11.42%)	
Mexican American	996 (15.77%)	803 (16.26%)	193 (14.04%)	
Other Hispanic	669 (10.59%)	521 (10.55%)	148 (10.76%)	
Other races	369 (5.84%)	306 (6.19%)	63 (4.58%)	
**Education level, n (%)**				0.799
<High school	1,728 (27.36%)	1,344 (27.21%)	384 (27.93%)	
High school	1,475 (23.36%)	1,151 (23.30%)	324 (23.56%)	
>High school	3,112 (49.28%)	2,445 (49.49%)	667 (48.51%)	
**Marital status, n (%)**				0.002
Married/Living with a partner	3,871 (61.30%)	2,999 (60.71%)	872 (63.42%)	
Divorced/Separated/Widowed	1,394 (22.07%)	1,077 (21.80%)	317 (23.05%)	
Never married	1,050 (16.63%)	864 (17.49%)	186 (13.53%)	
**UIC, n (%)**				<0.001
<100 ug/L	2,007 (31.78%)	1,615 (32.69%)	392 (28.51%)	
100-300 ug/L	3,078 (48.74%)	2,407 (48.72%)	671 (48.80%)	
≥300 ug/L	1,230 (19.48%)	918 (18.58%)	312 (22.69%)	
**Diabetes, n (%)**				<0.001
No	5,133 (81.28%)	4,068 (82.35%)	1,065 (77.45%)	
Yes	1,182 (18.72%)	872 (17.65%)	310 (22.55%)	
**Hypertension, n (%)**				<0.001
No	3,554 (56.28%)	2,897 (58.64%)	657 (47.78%)	
Yes	2,761 (43.72%)	2,043 (41.36%)	718 (52.22%)	
**Cardiovascular diseases, n (%)**				<0.001
No	5,807 (91.96%)	4,575 (92.61%)	1,232 (89.60%)	
Yes	508 (8.04%)	365 (7.39%)	143 (10.40%)	
**Congestive heart failure, n (%)**				0.030
No	6,127 (97.02%)	4,805 (97.27%)	1,322 (96.15%)	
Yes	188 (2.98%)	135 (2.73%)	53 (3.85%)	
**Coronary heart disease, n (%)**				<0.001
No	6,075 (96.20%)	4,773 (96.62%)	1,302 (94.69%)	
Yes	240 (3.80%)	167 (3.38%)	73 (5.31%)	
**Angina pectoris, n (%)**				0.485
No	6,170 (97.70%)	4,830 (97.77%)	1,340 (97.45%)	
Yes	145 (2.30%)	110 (2.23%)	35 (2.55%)	
**Heart attack, n (%)**				0.005
No	6,042 (95.68%)	4,745 (96.05%)	1,297 (94.33%)	
Yes	273 (4.32%)	195 (3.95%)	78 (5.67%)	
**BMI, kg/m2**	28.96 ± 6.53	28.78 ± 6.36	29.60 ± 7.05	<0.001
**TC, mg/dL**	195.72 ± 41.19	194.99 ± 40.93	198.34 ± 42.01	0.008
**HDL, mg/dL**	52.06 ± 15.64	52.35 ± 15.65	50.99 ± 15.58	0.004
**FT4, pmol/L**	0.80 (0.70, 0.90)	0.80 (0.70, 0.90)	0.80 (0.70, 0.88)	<0.001
**TSH, mIU/L**	1.56 (1.04, 2.35)	1.32 (0.93, 1.77)	3.30 (2.82, 4.17)	<0.001
**LE8 score**	64.80 ± 14.94	65.14 ± 14.95	63.55 ± 14.86	<0.001
**Health behaviors score**	63.71 ± 20.55	63.46 ± 20.61	64.61 ± 20.33	0.068
HEI-2015 diet score	39.77 ± 31.34	38.78 ± 31.11	43.31 ± 31.90	<0.001
Physical activity score	69.15 ± 43.54	70.90 ± 42.80	62.86 ± 45.56	<0.001
Nicotine exposure score	65.87 ± 41.47	64.49 ± 42.09	70.82 ± 38.74	<0.001
Sleep health score	80.07 ± 26.33	79.69 ± 26.48	81.43 ± 25.72	0.030
**Health factors score**	65.88 ± 19.96	66.82 ± 20.02	62.50 ± 19.39	<0.001
Body mass index score	60.58 ± 33.07	61.38 ± 32.79	57.67 ± 33.93	<0.001
Blood lipids score	62.41 ± 31.21	63.28 ± 31.36	59.30 ± 30.44	<0.001
Blood glucose score	78.16 ± 27.72	78.85 ± 27.52	75.67 ± 28.28	<0.001
Blood pressure score	62.39 ± 34.56	63.78 ± 34.19	57.36 ± 35.44	<0.001
**CVH, n (%)**				0.037
Low	1,031 (16.33%)	782 (15.83%)	249 (18.11%)	
Moderate	4,204 (66.57%)	3,289 (66.58%)	915 (66.55%)	
High	1,080 (17.10%)	869 (17.59%)	211 (15.35%)	

Strict-normal thyroid function was defined as plasma TSH of 0.34-2.5 mIU/L and normal FT4 level. Low thyroid function was defined as a serum TSH level over 2.5 mIU/L and a normal FT4 level, including both low-normal thyroid function and subclinical hypothyroidism.

Low CVH was defined as a LE8 score of 0 to 49, moderate CVH of 50-79, and high CVH of 80-100.

UIC, urine iodine concentration; BMI, body mass index; TC, total cholesterol; HDL, high density lipoprotein; FT4, free thyroxine; TSH, thyroid-stimulating hormone; LE8, Life’s Essential 8; HEI, healthy eating index; CVH, cardiovascular health.

### 3.2 Association between LE8 and its components with low thyroid function

**Table 2** demonstrated the findings of the multivariate regression analyses between the LE8 score and low thyroid function. The completely adjusted model suggested that the high CVH group (OR: 0.757, 95%CI: 0.607-0.944) had a considerably lower probability of low thyroid function in comparison with the low. For every 10-point increase in LE8 score, the risk of developing low thyroid function was reduced by 7.7% (OR: 0.923, 95%CI: 0.884-0.964). However, the health behaviors score did not substantially correlate with low thyroid function in multivariate regression analysis (P>0.05). As for the health factors score, fully adjusted models revealed a significantly lower risk of developing low thyroid function in the moderate (OR: 0.789, 95%CI: 0.680-0.917) and the high (OR: 0.572, 95%CI: 0.475-0.688) health factors groups in comparison to the low. For every 10-point increase in the health factor score, the risk of developing low thyroid function was reduced by 9.5% (OR: 0.905, 95% CI: 0.876-0.935).

**Table 2:** Association between the LE8 score and low thyroid function.

	Model 1^a^		Model 2^b^		Model 3^c^	
	OR(95% CI)	*P*-value	OR(95% CI)	*P*-value	OR(95% CI)	*P*-value
LE8 score						
Low (0-49)	1.000 (Reference)		1.000 (Reference)		1.000 (Reference)	
Moderate (50-79)	0.874 (0.744, 1.026)	0.09885	0.862 (0.732, 1.016)	0.07696	0.856 (0.725, 1.011)	0.06705
High (80-100)	0.763 (0.620, 0.938)	0.01037	0.767 (0.619, 0.950)	0.01496	0.757 (0.607, 0.944)	0.01341
Per 10-point increase	0.932 (0.895, 0.969)	0.00049	0.928 (0.890, 0.968)	0.00044	0.923 (0.884, 0.964)	0.00030
Health behaviors score						
Low (0-49)	1.000 (Reference)		1.000 (Reference)		1.000 (Reference)	
Moderate (50-79)	1.092 (0.939, 1.271)	0.25158	1.049 (0.900, 1.224)	0.53907	1.047 (0.896, 1.223)	0.56591
High (80-100)	1.108 (0.938, 1.310)	0.22837	1.004 (0.846, 1.191)	0.96340	1.003 (0.841, 1.197)	0.97102
Per 10-point increase	1.028 (0.998, 1.058)	0.06834	1.008 (0.979, 1.039)	0.58871	1.009 (0.978, 1.041)	0.56742
Health factors score						
Low (0-49)	1.000 (Reference)		1.000 (Reference)		1.000 (Reference)	
Moderate (50-79)	0.794 (0.686, 0.918)	0.00181	0.789 (0.680, 0.916)	0.00186	0.789 (0.680, 0.917)	0.00196
High (80-100)	0.533 (0.448, 0.634)	<0.00001	0.578 (0.482, 0.693)	<0.00001	0.572 (0.475, 0.688)	<0.00001
Per 10-point increase	0.897 (0.871, 0.925)	<0.00001	0.907 (0.879, 0.937)	<0.00001	0.905 (0.876, 0.935)	<0.00001

^a^Model 1: no covariates were adjusted.

^b^Model 2: age strata, sex, and race/ethnicity were adjusted.

^c^Model 3: age strata, sex, race/ethnicity, education level, marital status, and UIC.

LE8, Life’s Essential 8; UIC, urine iodine concentration; OR, odds ratio; CI, confidence interval.

**Figure 2:**
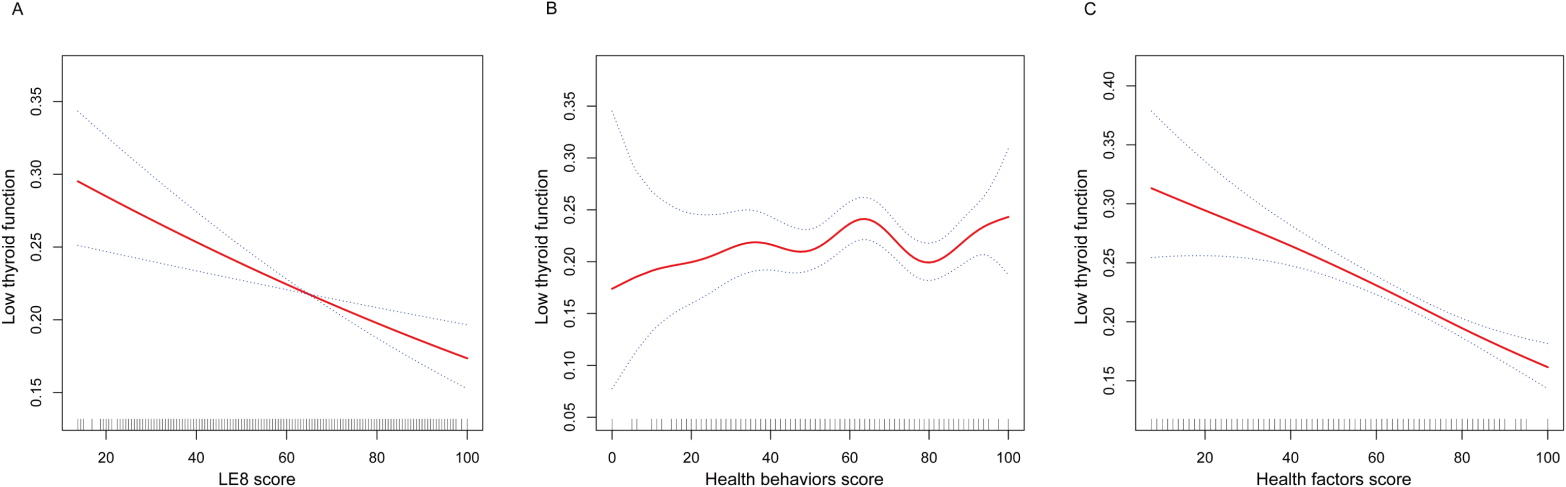
The nonlinear associations between LE8 and its subscales with low thyroid function. **(A)** The nonlinear associations between LE8 score and low thyroid function. **(B)** The nonlinear associations between Health behaviors score and low thyroid function. **(C)** The nonlinear associations between Health factors score and low thyroid function. LE8, Life’s Essential 8.

The nonlinear associations between LE8, health behaviors, and health factors with low thyroid function were presented in **Figure 2**. The results showed a linear negative relationship between LE8 and health factors and low thyroid function, and there was no significant correlation between health behaviors and low thyroid function.

Moreover, the outcomes of the multivariate regression analyses between components of LE8 and low thyroid function were displayed in **Table 3**. Fully adjusted models suggested that among the LE8 components, elevated scores in PA, BMI, blood lipid, BG, and BP might have favorable effects on diminishing the incidence of low thyroid function.

**Table 3:** Associations between the components of LE8 and low thyroid function.

Components of LE8	Cases/participants	OR(95%CI)	*P*-value
HEI-2015 diet score			
Low (0-49)	618/3,157	1.000 (Reference)	
Moderate (50-79)	349/1,578	1.126 (0.967, 1.311)	0.12535
High (80-100)	408/1,580	1.245 (1.070, 1.449)	0.00457
Per 10-point increase	1,375/6,315	1.260 (1.083, 1.465)	0.00279
Physical activity score			
Low (0-49)	497/1,875	1.000 (Reference)	
Moderate (50-79)	58/223	1.005 (0.727, 1.388)	0.97776
High (80-100)	820/4,217	0.705 (0.615, 0.807)	<0.00001
Per 10-point increase	1,375/6,315	0.965 (0.952, 0.979)	<0.00001
Nicotine exposure score			
Low (0-49)	303/1,725	1.000 (Reference)	
Moderate (50-79)	325/1,367	1.146 (0.949, 1.384)	0.15700
High (80-100)	747/3,223	1.359 (1.159, 1.593)	0.00016
Per 10-point increase	1,375/6,315	1.033 (1.016, 1.050)	0.00011
Sleep health score			
Low (0-49)	239/1,159	1.000 (Reference)	
Moderate (50-79)	290/1,480	0.920 (0.756, 1.119)	0.40373
High (80-100)	846/3,676	1.032 (0.873, 1.220)	0.70914
Per 10-point increase	1,375/6,315	1.009 (0.985, 1.033)	0.47804
Body mass index score			
Low (0-49)	564/2,333	1.000 (Reference)	
Moderate (50-79)	446/2,176	0.755 (0.653, 0.873)	0.00014
High (80-100)	365/1,806	0.750 (0.643, 0.876)	0.00027
Per 10-point increase	1,375/6,315	0.957 (0.939, 0.975)	<0.00001
Blood lipids score			
Low (0-49)	549/2,310	1.000 (Reference)	
Moderate (50-79)	333/1,472	0.996 (0.850, 1.167)	0.96510
High (80-100)	493/2,533	0.778 (0.675, 0.896)	0.00049
Per 10-point increase	1,375/6,315	0.964 (0.945, 0.983)	0.00022
Blood glucose score			
Low (0-49)	310/1,182	1.000 (Reference)	
Moderate (50-79)	322/1,457	0.838 (0.697, 1.007) 0.05973	0.06973
High (80-100)	743/3,676	0.779 (0.660, 0.919) 0.00308	0.01094
Per 10-point increase	1,375/6,315	0.970 (0.948, 0.992)	0.00908
Blood pressure score			
Low (0-49)	485/1,814	1.000 (Reference)	
Moderate (50-79)	408/2,004	0.775 (0.662, 0.909)	0.00167
High (80-100)	482/2,497	0.742 (0.634, 0.869)	0.00021
Per 10-point increase	1,375/6,315	0.965 (0.947, 0.983)	0.00020

ORs and 95% CIs were adjusted for age strata, sex, race/ethnicity, education level, marital status, and UIC.

LE8, Life’s Essential 8; UIC, urine iodine concentration; OR, odds ratio; CI, confidence interval.

### 3.3 Subgroup and sensitivity analysis

**Figure 3:**
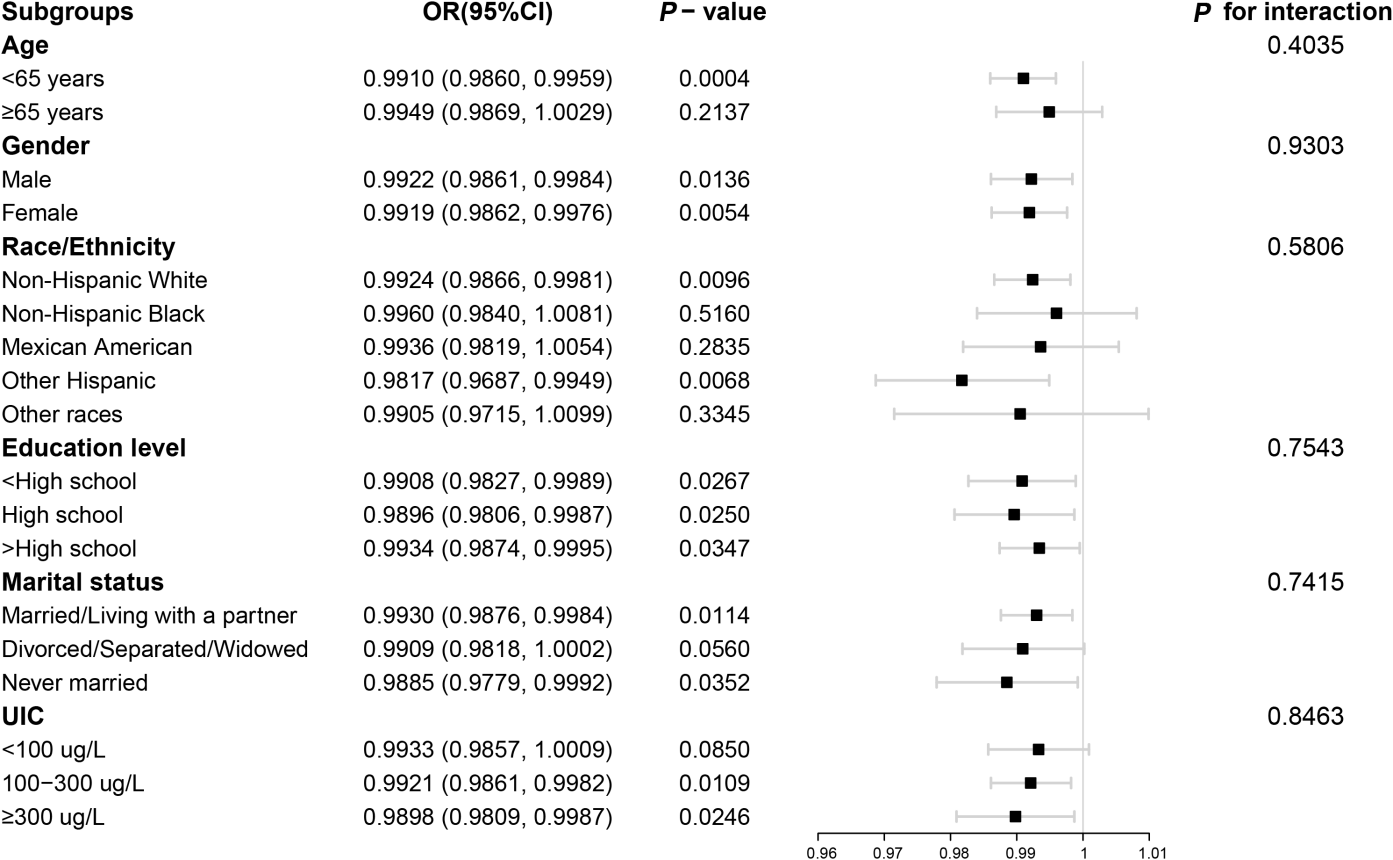
Subgroup analysis for the association between LE8 and low thyroid function. LE8, Life’s Essential 8; UIC, urine iodine concentration.

**Figure 4:**
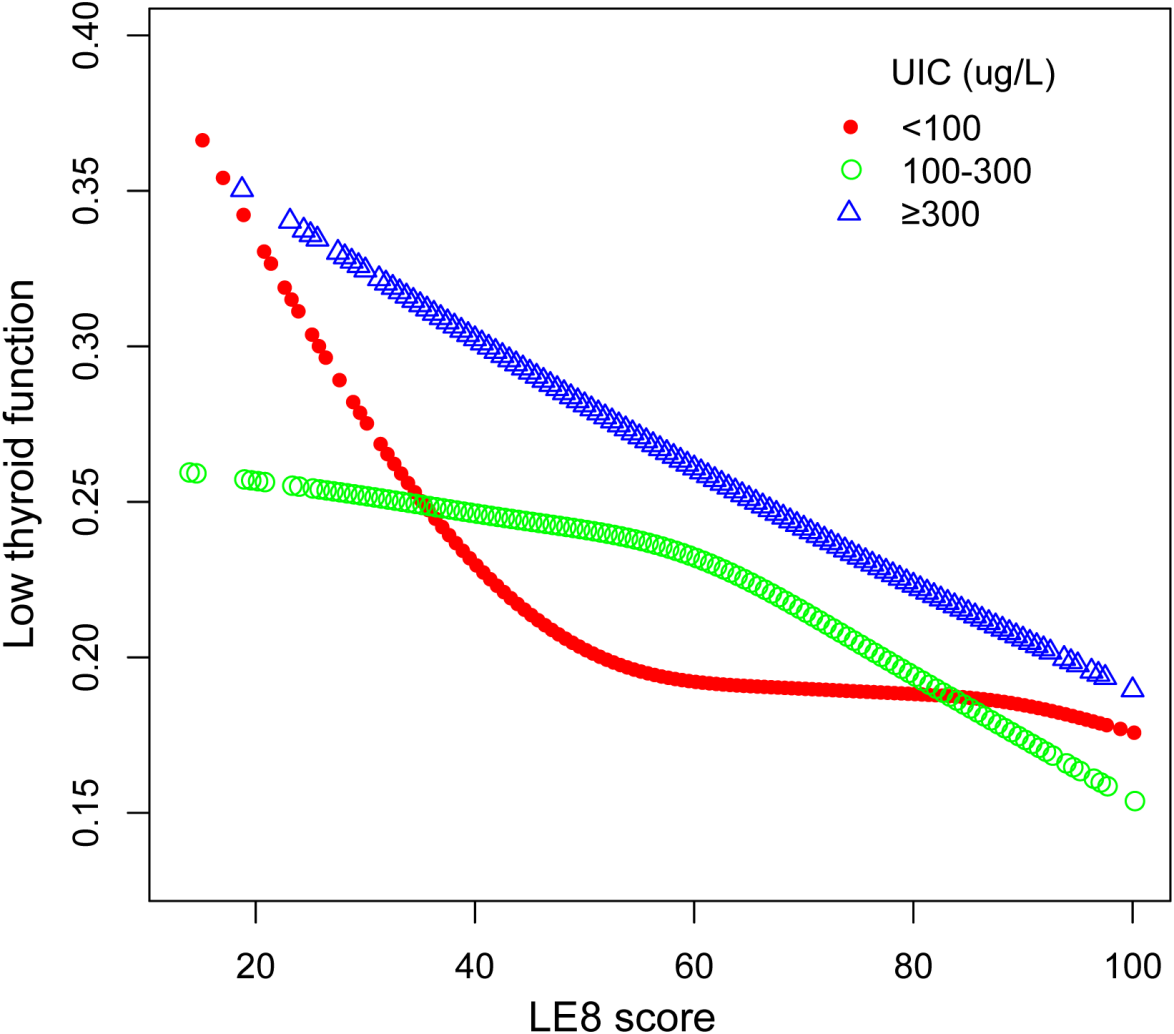
The association between LE8 and low thyroid function stratified by UIC. LE8, Life’s Essential 8; UIC, urine iodine concentration.

The outcomes of the subgroup analyses in **Figure 3** illustrated that LE8 was negatively linked to low thyroid function in various subpopulations, which was in agreement with the preliminary findings. Among them, the connection between LE8 and low thyroid function categorized by UIC was displayed in **Figure 4**. In the UIC<100 ug/L group, an L-shaped curve with an inflection point of 41.25 could be obtained after threshold calculation (**Supplementary Table 3**). When the LE8 score was below the inflection point, a higher LE8 score manifested a significant correlation with a lower risk of low thyroid function (P<0.05), and were not significant when LE8 scores were above the inflection point. Additionally, in the 100 ≤ UIC < 300 ug/L group, an L-shaped curve with an inflection point of 60 was obtained after threshold calculation (**Supplementary Table 3**). When the LE8 score was higher than the inflection point, a higher LE8 score performed a significant connection with a lower risk of low thyroid function (P<0.05), and were not significant when LE8 scores were below the inflection point. While in the 300 ug/L≤ UIC group, a curve with an inflection point of 38.13 was obtained after calculating the threshold (**Supplementary Table 3**). When the LE8 score was higher than the inflection point, a higher LE8 score performed a significant connection with a lower risk of low thyroid function (P<0.05).

Finally, the results remained robust after removing those who had histories of CVDs in the sensitivity analyses (**Table 4**).

**Table 4:** Sensitivity analysis of the association of the LE8 score with low thyroid function.

	Excluding participants with CVD history		
	Cases/participants	OR(95%CI)	*P*-value
LE8 score			
Low (0-49)	200/869	1.00 (Reference)	
Moderate (50-79)	831/3,887	0.891 (0.743, 1.068)	0.21094
High (80-100)	201/1,051	0.777 (0.615, 0.982)	0.03445
Per 10-point increase	1,232/5,807	0.927 (0.885, 0.971)	0.00126
Health behaviors score			
Low (0-49)	268/1,351	1.00 (Reference)	
Moderate (50-79)	603/2,799	1.058 (0.896, 1.249)	0.50431
High (80-100)	361/1,657	1.015 (0.842, 1.223)	0.87725
Per 10-point increase	1,232/5,807	1.001 (0.998, 1.005)	0.37728
Health factors score			
Low (0-49)	320/1,203	1.00 (Reference)	
Moderate (50-79)	639/2,906	0.779 (0.664, 0.914)	0.00225
High (80-100)	273/1,698	0.565 (0.465, 0.686)	<0.00001
Per 10-point increase	1,232/5,807	0.990 (0.987, 0.993)	<0.00001

ORs and 95% CIs were adjusted for age strata, sex, race/ethnicity, education level, marital status, and UIC.

LE8, Life’s Essential 8; UIC, urine iodine concentration; OR, odds ratio; CI, confidence interval.

## 4 Discussion

This nationwide investigation discovered that the higher LE8 score and the health factors score were related to a reduced hazard of low thyroid function in US adults. Meanwhile, higher scores in PA, BMI, blood lipid, BG, and BP would probably be favorable in terms of a lower incidence of low thyroid function. Subgroup and sensitivity analyses illustrated that the negative relationships were robust overall.

To our knowledge, our research investigated the connection between CVH, which was quantified by the LE8 score, and low thyroid function for the first time. A couple of earlier studies have already discussed the involvement between CVD and low thyroid function. Kosuke et al. discovered that CVDs acted as a mediator connecting SCH and high-normal TSH levels with all-cause mortality among US adults, especially in women and the elderly, suggesting possible threats to the health of patients even with only mildly elevated TSH levels (22). A study anchored in NHANES demonstrated that low thyroid function was related to nonalcoholic fatty liver disease (NAFLD) and was also a separate hazard indicator for heightened all-cause and cardiovascular mortalities in persons suffering from NAFLD (13). Yu-ling et al. also reported a similar negative Impact of low thyroid function on all-cause and cardiovascular deaths among metabolic dysfunction-associated fatty liver disease (MAFLD) populations, emphasizing the significance of reassessing the TSH reference range (23). Anna et al. discovered that among 744 women with normal thyroid function, TSH values at the upper end of the recommended range were linked to worse cardiometabolic profiles, as evidenced by a larger waist circumference, elevated values of BP, total cholesterol (TC), triglycerides (TG), and BG, and decreased values of high-density lipoprotein (HDL-C) (24). A cohort study of diabetic individuals from the US reviewed that high-normal TSH levels were linked to a rise in CVD mortality (25). Another proof gathered through a multi-cohort Mendelian randomization and metabolomics analyses revealed that TSH at the upper limit of the standard range led to poor blood lipid profiles and a higher incidence of CVDs (26).

The current research presented further evidence supporting the relationship between CVH and low thyroid function utilizing an updated CVH evaluation index. We observed that the LE8 score and the health factors score had a negative correlation with low thyroid function, confirming the previous reports. Also, intensive management of PA, BMI, blood lipids, BG, and BP is of vital importance in controlling the incidence of low thyroid function. An investigation noted that women with SCH had a remarkable reduction in PA duration, steps taken, grip and quadriceps strength, and functional motor ability in comparison to the healthy control subjects (27). The result of a Mendelian randomization revealed that obesity was one of the risk factors for hypothyroidism, and individuals with higher BMI had a heightened threat of developing hypothyroidism (28). Another meta-analysis concluded that SCH appeared to be connected to an elevated hazard of metabolic syndrome components such as obesity, hypertension, high TG values, and low HDL-C values (29). Besides, a prospective study of 72,003 participants found that after controlling for risk factors, individuals with high-normal TSH values experienced a 15% greater chance of developing prediabetes (30). In addition, the consequences of our subgroup analyses revealed that the negative correlation between the LE8 score and low thyroid function was more pronounced in women under 65 years of age and unmarried participants, suggesting that enhanced monitoring of CVD and thyroid function in such populations might have long-term benefits. Moreover, for the population with UIC in the normal range (100 ≤ UIC < 300 ug/L), the OR correlated between the LE8 score and low thyroid function declined smoothly in the lower part of the score and sharply in the higher part, which implied that more stringent CVH criteria were likely to be desirable for the generalized population. It was worth pointing out that in this study, we defined the TSH normal range more strictly, and the findings also showed that appropriately decreasing the maximum limit of the TSH benchmark range would be advantageous for identifying individuals at an increased potential risk of CVDs.

It is hypothesized that there are a couple of potential explanations for the relationship between CVD and low thyroid function. Higher levels of TSH are related to many CVD risk elements such as dyslipidemia, hypertension, diminished myocardial systolic and diastolic function, endothelial dysfunction, insulin resistance, etc. (2, 6). TSH can regulate lipid metabolism by combining with specific TSH receptors on the surface of hepatocytes and adipocytes, which primarily leads to raised levels of proprotein convertase subtilisin/kexin type 9 (PCSK9), liver 3-hydroxy-3-methyl glutaryl coenzyme A (HMG-COA) reductase (HMGCR), and hormone-sensitive lipase (HSL), as well as reduced levels of cholesterol 7α-hydroxylase (CYP7A1), inducing cholesterol accumulation and inhibiting clearance (31). Meanwhile, low thyroid function can result in decreased cardiac systolic and diastolic function by modulating the expression of contractile proteins and calcium uptake in cardiomyocytes (3). Furthermore, higher levels of TSH are also likely to promote endothelial dysfunction by augmenting endothelin (ET-1) levels and decreasing nitric oxide (NO) levels, which can contribute to atherosclerosis, increased peripheral vascular resistance, and raised blood pressure (32, 33). What’s more, it has been described that low thyroid function can bring about obvious insulin resistance, and the latter interacts with hyperhomocysteinemia (HHcy), which may provoke vascular endothelial damage and interfere with the process of atherosclerosis and CVD by increasing oxidative stress, stimulating endoplasmic reticulum stress, affecting epigenetic modifications, and altering protein function (34, 35). The mentioned mechanisms provide partially convincing evidence for our study, while more definite principles are expected to be further explored in depth.

Our current investigation has certain limitations. Firstly, even after controlling for a few possible confounders, the cross-sectional character of the research prevented us from concluding a causal and longitudinal association between LE8 and low thyroid function, and we could not completely discard the risk of bias caused by other confounders. Therefore, prospective research with larger sample sizes and longitudinal measurements is warranted to validate our findings. Secondly, the evaluations of certain indicators in LE8 were questionnaire-based, which were susceptible to recall bias. What’s more, as the complex effects of childhood and pregnancy on thyroid function have not yet been validly determined, we excluded underage and pregnant participants, and further explorations for these populations are called for in the future. In addition, the active form of thyroid, triiodothyronine, was not included in our study, and its levels may have important implications for CVH. We plan to explore more comprehensive indicators of thyroid function, including triiodothyronine, in future studies. Nonetheless, our study has several strengths. We employed a sufficient amount of a nationally representative sample to make our results more generalizable. Subgroup and sensitivity analyses were also carried out to augment the reliability of our outcomes.

## 5 Conclusion

In conclusion, the LE8 score and health factors score were nonlinearly and negatively correlated with the prevalence of low thyroid function. Also, high scores in PA, BMI, blood lipids, BG, and BP assessments should emphasized in the LE8 components. This study suggested that elevated TSH levels might have an implication on CVH even with normal thyroid function. Therefore, the significance of reassessing the TSH reference range should be emphasized. LE8, which is a clinically feasible and comprehensive indicator for improving CVH, may help patients identify the risk of low thyroid function at an early stage and play a potentially meaningful role in the promotion of thyroid health.

## Data Availability

The datasets presented in this study can be found in online repositories. The names of the repository/repositories and accession number(s) can be found below: https://www.cdc.gov/nchs/nhanes/.
