# Supplementary material for: Associations between cardiovascular health and low thyroid function among US adults: a population-based study
Source: Front Endocrinol (Lausanne). 2024 Sep 27;15:1437386. doi: 10.3389/fendo.2024.1437386 (PMC11466827; doi:10.3389/fendo.2024.1437386)
Supplement: Supplementary file 1 [file Table1.docx]

Supplementary Material

# Supplementary Tables

**Supplementary Table 1.** Definition and scoring approach for the American Heart Association’s Life’s Essential 8 score.

| Domain | CVH metric | Method of measurement | Quantification of CVH metric | |
| --- | --- | --- | --- | --- |
| Health behaviors | Diet | Self-reported daily intake of a DASH-style eating pattern | Quantiles of Healthy Eating Index-2015 | |
|  |  |  | Scoring: | |
|  |  |  | Points | Quantile |
|  |  |  | 100 | ≥95th percentile (top/ideal diet) |
|  |  |  | 80 | 75th-94th percentile |
|  |  |  | 50 | 50th-74th percentile |
|  |  |  | 25 | 25th-49th percentile |
|  |  |  | 0 | 1st-24th percentile (bottom/least ideal quartile) |
|  | Physical activity | Self-reported minutes of moderate or vigorous physical activity per week | Metric: Minutes of moderate (or greater) intensity activity per week | |
|  |  |  | Scoring: | |
|  |  |  | Points | Minutes |
|  |  |  | 100 | ≥150 |
|  |  |  | 90 | 120-149 |
|  |  |  | 80 | 90-119 |
|  |  |  | 60 | 60-89 |
|  |  |  | 40 | 30-59 |
|  |  |  | 20 | 1-29 |
|  |  |  | 0 | 0 |
|  | Nicotine exposure | Self-reported use of cigarettes or inhaled nicotine-delivery system | Metric: Combustible tobacco use and/or inhaled NDS use; or secondhand smoke exposure | |
|  |  |  | Scoring: |  |
|  |  |  | Points | Status |
|  |  |  | 100 | Never smoker |
|  |  |  | 75 | Former smoker, quit ≥5 yrs |
|  |  |  | 50 | Former smoker, quit 1-<5 yrs |
|  |  |  | 25 | Former smoker, quit <1 year, or currently using inhaled NDS |
|  |  |  | 0 | Current smoker |
|  |  |  | Subtract 20 points (unless score is 0) for living with active indoor smoker in home | |
|  | Sleep health | Self-reported average hours of sleep per night | Metric: Average hours of sleep per night | |
|  |  |  | Scoring: | |
|  |  |  | Points | Level |
|  |  |  | 100 | 7-<9 |
|  |  |  | 90 | 9-<10 |
|  |  |  | 70 | 6-<7 |
|  |  |  | 40 | 5-<6 or ≥10 |
|  |  |  | 20 | 4-<5 |
|  |  |  | 0 | <4 |
| Health Factors | Body mass index | Body weight (kg) divided by height squared (m^2^) | Metric: Body mass index (kg/m^2^) | |
|  |  |  | Scoring: | |
|  |  |  | Points | Level |
|  |  |  | 100 | <25 |
|  |  |  | 70 | 25.0-29.9 |
|  |  |  | 30 | 30.0-34.9 |
|  |  |  | 15 | 35.0-39.9 |
|  |  |  | 0 | ≥40.0 |
|  | Blood lipids | Plasma total and HDL-cholesterol with calculation of non-HDL-cholesterol | Metric: Non-HDL-cholesterol (mg/dL) | |
|  |  |  | Scoring: | |
|  |  |  | Points | Level |
|  |  |  | 100 | <130 |
|  |  |  | 60 | 130-159 |
|  |  |  | 40 | 160-189 |
|  |  |  | 20 | 190-219 |
|  |  |  | 0 | ≥220 |
|  |  |  | If drug-treated level, subtract 20 points | |
|  | Blood glucose | Fasting blood glucose or casual hemoglobin A1c | Metric: Fasting blood glucose (mg/dL) or Hemoglobin A1c (%) | |
|  |  |  | Scoring: | |
|  |  |  | Points | Level |
|  |  |  | 100 | No history of diabetes and FBG <100 (or HbA1c < 5.7) |
|  |  |  | 60 | No diabetes and FBG 100-125 (or HbA1c 5.7-6.4) (Pre-diabetes) |
|  |  |  | 40 | Diabetes with HbA1c <7.0 |
|  |  |  | 30 | Diabetes with HbA1c 7.0-7.9 |
|  |  |  | 20 | Diabetes with HbA1c 8.0-8.9 |
|  |  |  | 10 | Diabetes with Hb A1c 9.0-9.9 |
|  |  |  | 0 | Diabetes with HbA1c ≥10.0 |
|  | Blood pressure | Appropriately measured systolic and diastolic blood pressure | Metric: Systolic and diastolic blood pressure (mmHg) | |
|  |  |  | Scoring: | |
|  |  |  | Points | Level |
|  |  |  | 100 | <120/<80 (Optimal) |
|  |  |  | 75 | 120-129/<80 (Elevated) |
|  |  |  | 50 | 130-139 or 80-89 (Stage I HTN) |
|  |  |  | 25 | 140-159 or 90-99 |
|  |  |  | 0 | ≥160 or ≥100 |
|  |  |  | Subtract 20 points if treated level | |

CVH, cardiovascular health; FBG, Fasting blood glucose; HbA1c, Hemoglobin A1c.

**Supplementary Table 2.** Definition and scoring approach for the American Heart Association’s Life’s Essential 8 score.

| Component | Maximum points | Standard for maximum score | Standard for minimum score of zero |
| --- | --- | --- | --- |
| Adequacy^†^ |  |  |  |
| Total Fruits | 5 | ≥0.8 cup equiv. per 1,000 kcal | No Fruit |
| Whole Fruits | 5 | ≥0.4 cup equiv. per 1,000 kcal | No Whole Fruit |
| Total Vegetables | 5 | ≥1.1 cup equiv. per 1,000 kcal | No Vegetables |
| Greens and Beans4 | 5 | ≥0.2 cup equiv. per 1,000 kcal | No Dark Green Vegetables or Legumes |
| Whole Grains | 10 | ≥1.5 oz equiv. per 1,000 kcal | No Whole Grains |
| Dairy | 10 | ≥1.3 cup equiv. per 1,000 kcal | No Dairy |
| Total Protein Foods | 5 | ≥2.5 oz equiv. per 1,000 kcal | No Protein Foods |
| Seafood and Plant Proteins | 5 | ≥0.8 oz equiv. per 1,000 kcal | No Seafood or Plant Proteins |
| Fatty Acids^‡^ | 10 | (PUFAs + MUFAs)/SFAs ≥2.5 | (PUFAs + MUFAs)/SFAs ≤1.2 |
| Moderation^§^ |  |  |  |
| Refined Grains | 10 | ≤1.8 oz equiv. per 1,000 kcal | ≥4.3 oz equiv. per 1,000 kcal |
| Sodium | 10 | ≤1.1 gram per 1,000 kcal | ≥2.0 grams per 1,000 kcal |
| Added Sugars | 10 | ≤6.5% of energy | ≥26% of energy |
| Saturated Fats | 10 | ≤8% of energy | ≥16% of energy |

*Intakes between the minimum and maximum standards are scored proportionately.

†Adequacy components represent the food groups, subgroups, and dietary elements that are encouraged. For these components, higher scores reflect higher intakes, because higher intakes are desirable.

‡Ratio of poly- and monounsaturated fatty acids (PUFAs and MUFAs) to saturated fatty acids (SFAs).

§Moderation components represent the food groups and dietary elements for which there are recommended limits to consumption. For moderation components, higher scores reflect lower intakes, because lower intakes are more desirable.

**Supplementary Table 3.** Threshold effect analysis of the LE8 score on low thyroid function using a linear regression model.

| **LE8 score** | **OR(95%CI)， *P*-value** |
| --- | --- |
| **UIC<100ug/L** |  |
| Inflection point | 41.25 |
| LE8<41.25 | 0.9354 (0.8907, 0.9824) 0.0076 |
| LE8≥41.25 | 0.9986 (0.9897, 1.0075) 0.7523 |
| Log likelihood ratio | 0.018 |
| **100ug/L≤UIC<300ug/L** |  |
| Inflection point | 60 |
| LE8<60 | 1.0047 (0.9909, 1.0187) 0.5082 |
| LE8≥60 | 0.9834 (0.9726, 0.9943) 0.0030 |
| Log likelihood ratio | 0.046 |
| **UIC≥300ug/L** |  |
| Inflection point | 38.125 |
| LE8<38.125 | 1.0329 (0.9496, 1.1235) 0.4506 |
| LE8≥38.125 | 0.9867 (0.9766, 0.9969) 0.0109 |
| Log likelihood ratio | 0.292 |

ORs and 95% CIs were adjusted for age strata, sex, race/ethnicity, education level, marital status, and UIC.

LE8, Life’s Essential 8; UIC, urine iodine concentration; OR, odds ratio; CI, confidence interval.
